# Is inflammatory micronucleation the key to a successful anti-mitotic cancer drug?

**DOI:** 10.1098/rsob.170182

**Published:** 2017-11-15

**Authors:** T. J. Mitchison, J. Pineda, J. Shi, S. Florian

**Affiliations:** 1 Department of Systems Biology, Harvard Medical School, Boston, MA, USA; 2 Hong Kong Baptist University, Kowloon, HK, Hong Kong

**Keywords:** paclitaxel, anti-mitotic, cancer, chemotherapy, kinesin-5, inflammation

## Abstract

Paclitaxel is a successful anti-cancer drug that kills cancer cells in two-dimensional culture through perturbation of mitosis, but whether it causes tumour regression by anti-mitotic actions is controversial. Drug candidates that specifically target mitosis, including inhibitors of kinesin-5, AurkA, AurkB and Plk1, disappointed in the clinic. Current explanations for this discrepancy include pharmacokinetic differences and hypothetical interphase actions of paclitaxel. Here, we discuss post-mitotic micronucleation as a special activity of taxanes that might explain their higher activity in solid tumours. We review data showing that cells which exit mitosis in paclitaxel are highly micronucleated and suffer post-mitotic DNA damage, and that these effects are much stronger for paclitaxel than kinesin-5 inhibitors. We propose that post-mitotic micronucleation promotes inflammatory signalling via cGAS–STING and other pathways. In tumours, this signalling may recruit cytotoxic leucocytes, damage blood vessels and prime T-cell responses, leading to whole-tumour regression. We discuss experiments that are needed to test the micronucleation hypothesis, and its implications for novel anti-mitotic targets and enhancement of taxane-based therapies.

## 1. Introduction

From the early 1990s to the mid-2010s, the mitosis field engaged in a grand experiment, to identify proteins other than tubulin that are essential for mitosis in human cells, develop clinical grade small molecule inhibitors and test them for anti-cancer action in man. The hope was for broad-spectrum anti-cancer drugs lacking the neurotoxicity of taxanes. The first two arms were highly successful, and the field is now blessed with potent and specific inhibitors of several essential mitosis proteins including two kinesins (kinesin-5, CenpE) and three kinases (AurkA, AurkB and Plk1). The clinical arm was much less successful. Most compounds lacked efficacy at the toxicity limit, which was mostly set by neutropenia or gut toxicity [1–3]. This clinical failure, across multiple mitosis-specific targets, sharply decreased pharmaceutical company interest in targeting mitosis. Its causes have been reviewed, with different authors focusing on the low proliferation rate in cancer cells [4,5] or differential pharmacodynamics (PD) [6].

The clinical failure of mitosis-specific drugs tested to date stands in stark contrast with the success of drugs that target microtubules, which include vinca alkaloids, taxanes, ixabepilone and eribulin. All these drugs perturb mitosis as their primary cytotoxic action in two-dimensional tissue culture, but whether this is their therapeutic action in patients is controversial [4–6]. The purpose of this review is to advance a hypothesis that is new to our knowledge, that the success of taxanes in solid tumour treatment is due to their ability to induce multiple micronuclei in cells that pass through mitosis in the presence of drug, which we will term ‘micronucleation’. The mitosis-specific drugs tested to date exhibit weaker micronucleation activity, which might explain their clinical failure.

**Figure 1.**
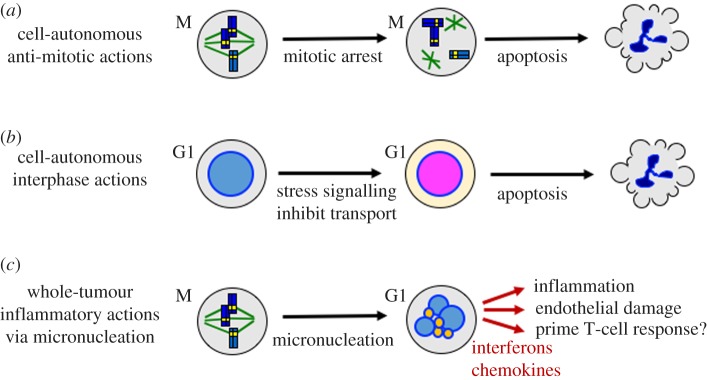
Three models for the anti-tumour actions of paclitaxel (illustration by T.J.M.). (*a*) Anti-mitotic actions that cause cell-autonomous death via mitotic arrest or chromosome mis-segregation. This well-characterized action accounts for essentially all cell killing in two-dimensional cell culture and is shared with mitosis-specific drugs [16,17]. (*b*) Interphase actions that cause cell-autonomous death of non-dividing cells. Proposed pathways include activation of MAPK signalling [18,19] and inhibition of cytoplasm–nucleus trafficking [20]. Mitosis-specific drugs lack these actions. (*c*) New model for whole-tumour action via inflammatory micronucleation. Perturbation of mitosis and cytokinesis by taxanes generates G1 cells with multiple micronuclei [7]. Small micronuclei are coloured orange to signify possible DNA damage [9] and cGAS recruitment. These effects in micronuclei activate inflammatory signalling, causing secretion of inflammatory cytokines and chemokines that promote whole-tumour regression in sensitive patients.

Micronuclei are small nuclei containing one or a few chromosomes that form due to mitotic chromosome segregation errors. Individual misplaced chromosomes recruit a nuclear envelope during telophase, and once formed, micronuclei typically do not fuse with the main nucleus. Paclitaxel induces dramatic segregation errors, leading to partitioning of the genome into many small nuclei that we term ‘micronucleation’. This effect was noted in early cytological studies of taxane action in two-dimensional culture, particularly at low drug concentrations that do not promote mitotic arrest [7,8]. We revisit it in the light of recent discoveries that micronuclei undergo dramatic DNA damage [9] and nuclear envelope rupture [10], and can activate the pro-inflammatory cGAS–STING pathway [11,12]. Pro-inflammatory signalling has the potential to cure tumours [13], and direct activation of the cGAS–STING pathway by small molecule STING agonists eliminates solid tumours in mice [14,15]. Putting all these data together, we propose that inflammatory signalling, caused by micronucleation, plays a central role in taxane action in tumours (figure 1*c*), and we will discuss this idea in detail below.

Tumour inflammation, and its role in therapy, is a hugely complex area that we will only touch on here. Our main goal is to draw attention to micronucleation as an underappreciated discriminating factor between taxanes and mitosis-specific drugs, and to review recent data on activation of inflammatory pathways by damage to the cell nucleus. We hope to open a new line of discussion around taxane action and potential novel cancer targets in mitosis. We emphasize that our model is speculative, that we respect alternative viewpoints and that more data are needed to decide these complex issues.

## 2. How do taxanes work in solid tumours?

Taxanes are among the most important drugs for solid tumour treatment, and their clinical activity is still being optimized. They bind to microtubules and stabilize the lattice, which inhibits dynamic instability and promotes ectopic nucleation. Two main models have been advanced to explain their anti-tumour activity. Figure 1*a* illustrates the standard anti-mitotic model, in which perturbation of mitosis causes cell-autonomous death of the dividing cell. This is the predominant action in tissue culture [6,21–23]. High taxane concentrations arrest dividing cells in mitosis by preventing silencing of the spindle assembly checkpoint (SAC). Subsequent behaviour is highly variable between cell lines and individual cells. Arrested cells can die inside mitosis, or slip out by cyclin B degradation. After mitotic exit they can die, undergo senescence or re-enter the cell cycle progression. Mitotic arrest is not necessary for mitosis-dependent cell death. Paclitaxel promotes chromosome mis-segregation at concentrations too low to cause mitotic arrest, which can lead to late cell death [7,8]. This action might be particularly relevant in tumours as the drug concentration decreases over time following a dose [24]. The clinical relevance of the anti-mitotic model was strongly criticized from the perspective that the slow proliferation rate in solid tumours is inconsistent with any model where only cells that divide in drug are killed [4].

Figure 1*b* illustrates the main alternative proposed by Fojo and others. In this model, taxanes act on interphase cells, e.g. to perturb nucleus–cytoplasm trafficking [20,25] or MAPK signalling [18,19], and this leads to cell-autonomous death. This interphase killing model naturally accounts for the clinical difference between taxanes and mitosis-specific drugs. The main deficiency we see with this model is the lack of definitive experimental systems where stabilization of interphase microtubules causes death of non-dividing cancer cells. Many studies have investigated alternative mechanisms of cell killing in two-dimensional cultures, but most are clouded by failure to critically discriminate mitosis-dependent versus -independent actions. Time-lapse imaging is arguably the best way to do this [16].

Figure 1*c* proposes an alternative ‘inflammatory micronucleation’ model that is new to our knowledge, though related to other recent proposals [22,24]. It highlights the well known, but underappreciated, mitosis-dependent micronucleating activity of taxanes, and proposes inflammatory signalling to amplify signals from a small fraction of dividing cells to eliminate the whole tumour. It makes multiple untested assumptions, and must be considered speculative. The central propositions of this model are: (i) *micronucleation* is a special action of taxanes and epothilones on dividing cells that is not shared with current mitosis-specific drugs, (ii) micronucleation promotes *inflammatory signalling* and (iii) inflammatory signalling from a subset of cells that pass through mitosis in drug promotes *whole-tumour regression*, at least in sensitive patients. Micronucleation can occur at taxane concentrations well below those required for mitotic arrest, and micronucleated cells can persist for days. These effects will likely lead to gradual accumulation of multi-nucleated cells in a drug-treated tumour, unlike apoptotic cells, which are rapidly cleared by phagocytosis. Below, we will review evidence that leads us to propose the micronucleation model, suggest experiments to test it and briefly discuss implications for novel anti-mitotic targets and taxane pharmacology.

## 3. Why did current mitosis-specific drugs fail in the clinic?

All available data on drugs that inhibit kinesin-5, CenpE, AurkA, AurkB and Plk1 attest that they can cause lethal perturbation of cell division by direct anti-mitotic actions (figure 1*a*). If they were present in patients at sufficient concentration for sufficient time, they should eliminate tumours by this mechanism. The problem is therapeutic index. All these proteins are equally required for mitosis in normal cells, and cell division occurs more frequently in the bone marrow and gut than in solid tumours [4,5]. Promyelocytes in the bone marrow are the fastest dividing cell population in adults, which likely explains why neutropenia was the dose-limiting toxicity of many of the drug candidates that specifically target mitosis [3]. The need to avoid bone marrow and gut toxicity presumably limited drug exposure in patients to below that required to cause tumour regression. Fojo and co-workers [4] argued that given the low proliferation rate in solid tumours compared to normal tissues, we cannot expect useful therapeutic index from purely anti-mitotic actions of any drug. This argument is strong, but it assumes that drug-induced cell death is cell-autonomous. We could rescue the anti-mitotic hypothesis if taxane action on mitosis causes extensive bystander killing, while the action of mitosis-specific drugs on mitosis does not [5].

The clinical failure of the mitosis-specific drugs raises serious criticisms of the assays that were used to advance them to the clinic. Fojo and co-workers [4] emphasized that two-dimensional cell cultures and mouse tumour models all exhibit cell division rates that are much higher than human tumours, and thus tend to over-predict the efficacy of drugs with cell cycle targets. We agree, but here we focus on a different issue, lack of measurements of inflammatory signalling during drug development. If solid tumours are eliminated by the consequences of this signalling, then measuring it in culture would better predict tumour responses than measuring cell death or inhibition of proliferation. Another criticism is that mouse studies appear to have systematically under-predicted the bone marrow toxicity of mitosis-specific drugs, and rats may provide a better model for this likely dose-limiting toxicity [26].

## 4. Paclitaxel and kinesin-5 inhibitors differ in post-mitotic micronucleation and DNA damage

It is instructive to compare the cellular actions of taxanes to mitosis-specific drugs in pre-clinical models to try and understand why paclitaxel is much more active as a solid tumour treatment in man. We will focus only on kinesin-5 inhibitors (K5Is) for simplicity. To what extent our arguments extend to inhibitors of mitotic kinases is an important open question. K5Is were the first mitosis-specific drugs to be tested in man, led by ispinesib from Cytokinetics/GSK [27]. K5I-treated cells arrest in mitosis with monopolar spindles [28], and most K5Is are extremely specific because they target allosteric pockets in the motor domain [29]. Our group and others directly compared the cellular actions of paclitaxel to K5Is in cell culture and mouse tumour models [17,30–33].

Differential cell killing during mitotic arrest is likely not a discriminator between paclitaxel and K5Is. Both drugs arrest cells in mitosis by preventing silencing of the same SAC. Arrested cells eventually exit mitosis by degradation of cyclin B without SAC inactivation, in a process termed mitotic slippage [34]. The duration of mitotic arrest and the fraction of arrested cells that die in mitosis versus those that slip out of mitosis were similar between paclitaxel and a K5I when the two drugs were compared at high concentrations that promoted strong mitotic arrest [17].

Interphase action is certainly a discriminator, though its clinical significance is unclear. Paclitaxel promotes stabilization and ectopic bundling of microtubules throughout the cell cycle, while the only known function of kinesin-5 (a.k.a. Kif11, Eg5, KSP) is to separate the poles of the mitotic spindle. The unresolved questions are if, and how, taxanes kill non-dividing cancer cells in tumours, and the extent to which interphase killing contributes to therapy. Potential interphase actions of paclitaxel were reviewed briefly above; the focus of this review is on mitosis-dependent actions.

Pharmacokinetics (PK) and PD may be discriminators. Paclitaxel tends to persist in solid tumours for many days, presumably bound to microtubules [6,35], while K5Is exhibit more ordinary pharmacology [1]. Paclitaxel causes chromosome segregation errors and micronucleation after mitotic exit at low concentrations that do not cause mitotic arrest [24,36]. These low-concentration effects, combined with prolonged residence of the drug in tumours, may well contribute to the higher clinical efficacy of taxanes.

**Figure 2.**
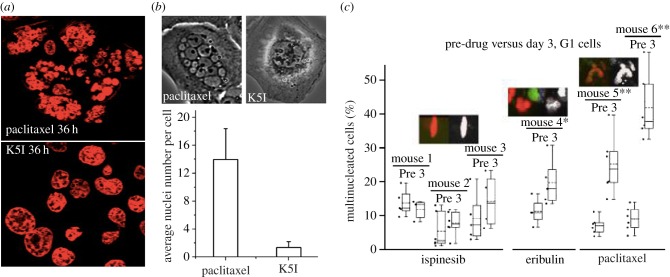
Nuclear morphology after exit from aberrant mitosis in paclitaxel versus K5Is. (*a*) DNA stained field of HT1888 cells in two-dimensional culture 36 h after treatment with paclitaxel (100 nM) or a K5I (K858, 10 µM). Most cells have slipped out of drug-arrested mitosis at this time point. Note multiple micronuclei in paclitaxel and mostly single nuclei in K5I. Taken from Nakai *et al*. [30]. (*b*) U2OS cells time-lapse imaged in two-dimensional culture in the presence of paclitaxel (150 nM) or a K5I (EMD534085 1 µM). Top panels are phase-contrast images of single cells that have slipped out of mitotic arrest after approximately 30 h in drug. The bar graph shows quantification of nuclei per post-slippage cell. Note multiple micronuclei in paclitaxel but not K5I. Taken from Zhu *et al*. [33]; *note senior author Shi is an author of the review.* (*c*) Quantification of nuclear morphology in HT1080 xenograft tumours by quantitative intravital microscopy before, and 72 h after, drug treatment. Nuclei were visualized with a transfected histone-CFP probe. Each pair of bars shows data from the same mouse before, and 3 days after, after a single drug dose. Ispinesib is a K5I, eribulin is a microtubule destabilizing drug. Paclitaxel caused extensive micronucleation, ispinesib very little and eribulin was intermediate. Separate data showed that the extent of mitotic arrest was similar in the three drugs, and that the microtubule drugs caused tumour regression, while the K5I did not. Taken from Chittajallu *et al*. [32].

Micronucleation is the discriminator we focus on in this review. As pointed out by Wilson and co-workers [7], cells that pass through a paclitaxel-inhibited mitosis in two-dimensional culture tend to exhibit multiple micronuclei, a morphology we refer to as ‘bunch of grapes’ nuclei (figure 2). In marked contrast, cells that slip out of K5I-inhibited mitosis tend to have a single large nucleus. Head-to-head comparison in two-dimensional culture and a mouse tumour model noted much stronger micronucleation after exit from mitosis in paclitaxel than a K5I (figure 2). For cytological analysis of micronucleation, it is important to distinguish it from apoptosis, because both cause a multi-lobed appearance of the nucleus. Chromatin is much more condensed during apoptosis, and apoptosis leads to cell rounding and blebbing shortly followed by lysis (in culture) or removal by phagocytosis (in tumours). Multi-nucleated cells, by contrast, decondense their chromatin and they can persist for days after exiting from a drug-treated mitosis.

**Figure 3.**
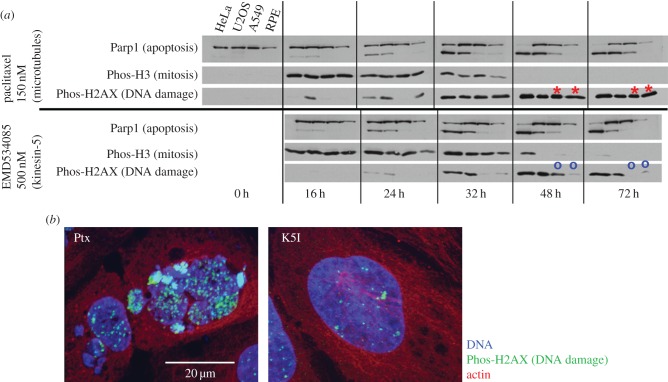
DNA damage signalling after slippage from drug-treated mitosis (*a*). Western blot analysis of apoptosis, mitosis and DNA damage in cells treated with paclitaxel or a K5I. Four cell lines growing in two-dimensional culture were treated with paclitaxel (150 nM) or EMD534085 (500 µM). Cells are arranged in order of apoptosis sensitivity, with HeLa most sensitive and RPE1 least. HeLa cells mostly died by apoptosis during mitotic arrest in both drugs. Note that apoptosis (scored by Parp1 cleavage) causes extensive DNA damage via the CAD pathway, so all conditions where Parp1 is cleaved also exhibit DNA damage signalling. A549 and RPE1 cells mostly slipped out of mitotic arrest without dying—note lack of Parp1 cleavage. In those lines, paclitaxel promoted extensive DNA damage signalling at 48 and 27 h (red asterisks), while K5I did not (blue circles). Modified from Shi *et al*. [17]; note *senior author Mitchison is an author of the review. This panel has been modified from the primary publication.* (*b*) Immunofluorescence of U2OS cells that have slipped out of mitotic arrest in paclitaxel versus K5I. The conditions are the same as figure 2*b*. Note strong H2AX staining in paclitaxel-induced micronuclei. The K5I-treated cell also exhibits some DNA damage, probably caused by CAD nuclease activity during prolonged mitotic arrest. Taken from Zhu *et al*. [33].

Post-slippage DNA damage is another potentially important discriminator that is mechanistically linked to micronucleation. Figure 3*a* shows data abstracted from papers where we measured markers of apoptosis, mitosis and DNA damage in parallel across four cell lines treated with saturating concentrations of paclitaxel versus a K5I [17]. The effects of the two drugs were very similar in lines that tend to undergo apoptosis during mitotic arrest or shortly afterwards (HeLa, U2OS). Note that apoptosis (marked by Parp1 cleavage) triggered strong DNA damage (marked by Phos-H2AX) due to DNA fragmentation by CAD nuclease. Here, we draw attention to extensive DNA damage that occurred in cells that exited mitosis in paclitaxel but *did not* initiate apoptosis, as is the case for A549 and RPE cells at 48 and 72 h (red asterisks in figure 3). This DNA damage signal was much stronger in paclitaxel than K5I (compare red asterisks to blue circles). At the time we did not understand its cause. In the light of new data on induction of DNA damage in micronuclei [9], we hypothesize this paclitaxel-specific, post-slippage, non-apoptotic DNA damage was caused by extensive micronucleation after slippage out of paclitaxel-treated mitosis, followed by micronucleus-triggered DNA damage. Consistent with this interpretation, figure 3*b* shows cytological evidence for DNA damage in a micronucleated cell from a more recent paper [33].

## 5. Paclitaxel micronucleation mechanism

**Figure 4.**
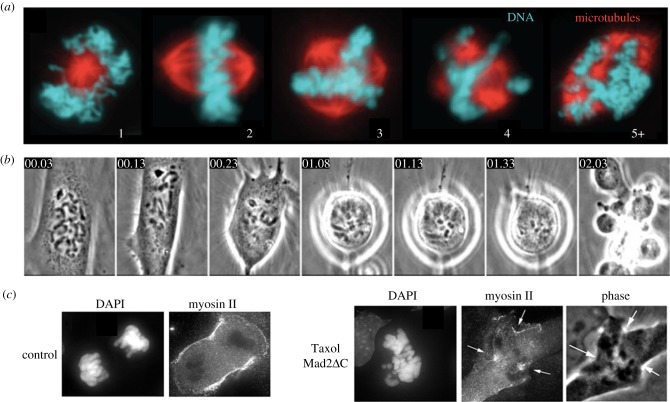
Paclitaxel promotes multi-polar spindles and ectopic cleavage furrows (*a*). Paclitaxel-treated mitotic MDA-MB-231 cells. Note multiple spindle poles in many cells. Numbers refer to spindle pole count. Spindles with three or more poles predominated at paclitaxel concentrations above 10 nM. Taken from Zasadil *et al*. [24]. (*b*) Time-lapse imaging of a dividing PtK cell in 10 µM paclitaxel. The SAC was slowly silenced in drug, and the cell progressed to cytokinesis. Note highly abnormal cleavage, with multiple ectopic furrows, in the last frame. Numbers refer to elapsed time (h : min). Taken from Yang *et al*. [37]. (*c*) Telophase PtK2 cells fixed and stained for DNA and myosin-II. In this experiment, the paclitaxel-treated cell (10 µM) was forced out of mitosis by inhibition of Mad2. Note formation of multiple ectopic furrows that recruit myosin-II (arrows). Taken from Shannon *et al*. [38].

The mechanisms by which paclitaxel promotes strong micronucleation have not been studied in detail, and warrant further analysis. We suspect at least two effects are at play based on published cytology (figure 4). Mitotic spindles tend to assemble with multiple poles in paclitaxel, even at low drug concentrations that do not promote strong mitotic arrest [24] (figure 4*a*). Ectopic poles are probably caused by ectopic microtubule nucleation, followed by clustering of minus ends by dynein and NUMA [39]. Kinesin-5 knockdown reduced micronucleation by paclitaxel, suggesting that kinesin-5 helps keep ectopic poles separated [33]. The presence of multiple spindle poles presumably leads to separation of chromosomes into multiple groups at anaphase onset. When paclitaxel-treated cells exit mitosis, they tend to exhibit ectopic cleavage furrows (figure 4*b,c*) [37,38]. These likely partition chromosome clusters into separate pockets of cytoplasm, keeping them separated during the critical period for nuclear envelope reformation in telophase. Ectopic furrows may result from recruitment of the furrow-stimulating complexes CPC and centralspindlin to Taxol-stabilized microtubule bundles [38,40]. Paclitaxel's combined effects of promoting multi-polar spindles and triggering ectopic cleavage furrows are not shared with current mitosis-specific drugs, and likely endow it with higher micronucleation activity.

## 6. DNA damage and nuclear envelope breakdown in micronuculei

Pioneering work from Pellman and co-workers [9] showed that the DNA in micronuclei formed by chromosome segregation errors tends to undergo extensive DNA damage, while the main nucleus in the same cell is spared. They went on to show this can account for the phenomenon of chromothripsis, where single chromosomes are fragmented and rearranged during cancer progression [41]. Separately, Hetzer and co-workers [10] showed that micronuclei tend to undergo catastrophic nuclear envelope breakdown that exposes their DNA to the cytoplasm. The relationship between these observations, and the mechanisms that induce them, is under intense investigation. These observations were made in cells with only one or a few micronuclei, but the data in figure 2 suggest that taxane-induced micronucleation also causes extensive DNA damage.

## 7. Inflammatory signalling in micronucleated cells

**Figure 5.**
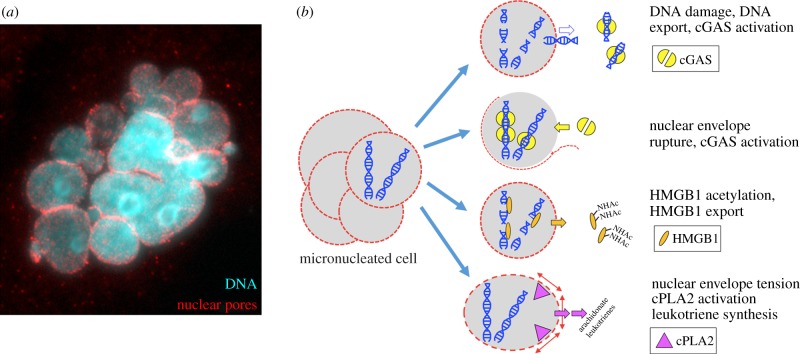
Candidate inflammatory pathways after micronucleation (illustration by T.J.M.). (*a*) Immunofluorescence image of a HeLa cell after passage though mitosis in paclitaxel showing multiple micronuclei and nuclear envelopes, in this cased stained for nuclear pores (T.J.M. 2007, unpublished data). (*b*) Four candidate pro-inflammatory pathways in micronucleated cells. See text for details.

Figure 5 illustrates four candidate inflammatory pathways that may be activated in micronucleated cells. This list is far from exclusive, and other pathways likely remain to be discovered. The top row in figure 5*b* illustrates nuclear export of DNA fragments following DNA damage [42,43]. Once exported, these fragments can bind to the cytosolic DNA sensor cGAS. DNA binding triggers synthesis of the second messenger 2′3′cGAMP which binds to STING [44]. STING then activates the TBK1–IRF3 pathway, leading to expression of inflammatory cytokines and chemokines including interferons. The second row illustrates cGAS activation by nuclear envelope collapse, which is relatively frequent in micronuclei [10]. This route to cGAS activation by micronuclei was recently implicated in the response to tumours to DNA damaging drugs and radiation [45,46]. Several other candidate nucleic acid sensors might also recognize DNA after nuclear export or/and nuclear envelope rupture [47]. The third row illustrates nuclear export of the chromatin protein HMGB1, which occurs via acetylation in response to DNA damage and other inflammatory triggers. Extracellular HMGB1 acts as an alarmin to activate inflammatory signalling [48], and was shown to promote anti-tumour inflammation in response to DNA damage in a mouse model [49]. Extracellular HMGB1 has also been implicated in response of human cancer to chemotherapy [50], so it is an important candidate factor when considering inflammatory responses to nuclear damage. The last row illustrates an interesting pathway by which tension in an intact nuclear envelope promotes cPLA2 and 5-lipoxygenase activation, leading to secretion of pro-inflammatory leukotrienes. This pathway was shown to mediate chemotaxis of leucocytes to wounds in zebrafish, where the trigger was osmotic swelling of nuclei at the wound margin [51]. Micronuclei might trigger cPLA2 activation and inflammatory leukotriene release if their envelope is under tension due to abnormally high surface area/volume ratios.

Secretion of inflammatory cytokines by micronucleated cells (figure 1*c*) conceptually resembles inflammatory signalling by senescent cells that have entered the ‘senescence-associated secretory phenotype’ (SASP) [22]. Data in recent papers suggest that this similarity may extend to causal mechanisms. Induction of senescence by DNA damage, oncogenes and ROS was shown to depend on cGAS–STING signalling [52–54]. The trigger in each case was blobs of chromatin in the cytoplasm of senescing cells that morphologically resemble micronuclei without nuclear envelopes, but are thought to form in a mitosis-independent manner, by extrusion from interphase nuclei. These chromatin blobs presumably signal in the same way as micronuclei whose envelopes have ruptured (figure 5*b*, second row). The morphological and signalling similarities between micronucleated and senescent cells revealed by these studies suggest that we might think of taxanes as drugs that promote mitosis-dependent SASP, as proposed by Cheng & Crasta [22].

## 8. From inflammation to whole-tumour responses

Inflammation can drive carcinogenesis and help tumours grow. Sufficiently strong inflammation can also destroy tumours. Which effects dominate under particular conditions is an intense current research topic. Untreated tumours are infiltrated with diverse leucocytes at baseline, to an extent that varies widely between patients [13]. Chemotherapy and radiation cause large changes in leucocyte infiltration and stromal inflammatory signalling which can oppose, or promote, therapy depending on the context [55]. Tumour-associated macrophages present at baseline tend to protect mouse tumours from taxanes [56]. In human breast tumours, paclitaxel caused a large influx of new macrophages, the extent of which correlated with response to therapy [57]. Taxane-induced leucocyte infiltration was also observed in a subset of mouse syngeneic tumour models, where it again correlated with strong responses [58]. Whether taxane-induced infiltrating leucocytes play a causal role in tumour regression, protect cancer cells or are merely cleaning up cell corpses killed by the drug is unknown.

The best evidence that strong inflammatory signalling can cause tumour regression comes from the therapeutic activity of directly pro-inflammatory anti-cancer drugs. For example, the TLR agonist imiquimod clears basal carcinomas in man via local inflammation [59], and the STING agonist DMXAA caused tumour regression in mice via vascular disruption and immune activation [60]. Whether taxane-treated human tumours contain sufficient multi-nucleated cells to drive a therapeutically useful, whole-tumour inflammatory response in an important open question.

## 9. Tumour selectivity

Taxane toxicities include neutropenia, alopecia, mucositis, dermatitis and peripheral neuropathy, so their actions are far from tumour-specific. However, they destroy solid tumours but not normal tissues in sensitive patients, so they do have a useful therapeutic index. None of the arguments above, or literature proposals we are aware of, explain this selectivity. Micronucleation alone does not help, because it should occur in normal dividing cells in the bone marrow and gut by the same mechanisms as in tumours, likely at a higher density due to the higher proliferation rate. Perhaps cell cycle checkpoints prevent progression to S-phase in micronucleated normal cells, but not in drug-sensitive cancer cells, resulting in more DNA damage-driven inflammatory signalling in tumours. Alternatively, inflammatory responses may be mis-regulated at the tissue-scale in tumours, e.g. tumour vasculature may be hyper-sensitive to inflammatory cytokines. In this case, similar inflammatory signalling by micronucleated cells would only cause tissue-scale destruction in tumours. The source of the therapeutic index in taxane chemotherapy is a crucial unanswered question.

## 10. Testing the inflammatory micronucleation model

The degree to which different anti-mitotic and microtubule-targeting drugs promote micronucleation after mitotic slippage has not, to our knowledge, been systematically measured or compared except for the K5I comparisons cited in figure 2. This should be straightforward in two-dimensional culture using high content assays, and is feasible in tumour models using histology and intravital microscopy [32]. We predict that taxanes promote more extensive micronucleation that any current mitosis-specific drugs. Comparisons between anti-microtubule drugs will also be interesting. Epothilone B (a stabilizing drug) cause more micronucleation than discodermolide (a destabilizer) in one study [8]. The microtubule destabilizing anti-cancer drug eribulin, which is approved for breast cancer treatment, was intermediate between paclitaxel and a K5I in promoting micronucleation in a mouse tumour model (figure 2*c*), but the number of mice sampled was too small for this to be a definitive conclusion.

A second important question is whether taxane-induced micronucleation activates inflammatory signalling in cancer cells, and if so, through which pathways. Obvious next steps are to measure inflammatory signalling in cells that have been micronucleated by taxanes, and test the effect of knocking out key pathway components such as cGAS, HMGB1 and cPLA2. An interesting possibility is that taxane-promoted interphase microtubule reorganization might synergize with micronucleation to promote inflammatory signalling. Comparison between drugs in these assays will require cell lines that do not undergo apoptosis during mitotic arrest or immediately after slippage, and may also depend on an intact cGAS–STING pathway, both of which vary across cancer cell lines.

We then need to know if taxanes promote inflammatory signalling in mouse tumour models, and if so, whether it is micronucleation-dependent, and whether it causes whole-tumour regression. A major challenge in this area is development of syngeneic or humanized models that accurately predict the immunobiology of human tumours, including response of leucocyte populations and tumour vasculature to drug perturbation [55].

Finally, and most important, will be to profile micronucleation-dependent inflammatory signalling in cancer cells from patients who do, and do not, respond to taxanes. As discussed below, selection against cGAS–STING-dependent inflammatory signalling during cancer progression may be a major factor shaping drug responses.

## 11. Novel targets in mitosis

Micronucleation is an unanticipated side effect of taxane action on microtubules. Do other targets exist for deliberately triggering micronucleation? One candidate is the VRK1–BAF pathway. BAF (a.k.a. BANF1) is a structural protein that mediates interaction between chromatin and nuclear envelope, and it is regulated by VRK1 kinase. Depletion of VRK1 or BAF promotes strong micronucleation of dividing cells [61,62]. It is possible that a VRK1 or BAF inhibitor would cause tumour regression via micronucleation as efficiently as paclitaxel, but lack neurotoxic side effects. Systematic investigation might reveal additional targets for micronucleation-promoting drugs.

## 12. Patient variation and drug resistance

Paclitaxel alone promotes tumour regression in approximately half of drug-naive breast cancer patients [63], and similar response variation is seen in other diseases. The cause of patient-to-patient variation is unknown despite decades of research. Relapsed disease is often refractory to further treatment, and the mechanistic basis of acquired resistance is also unclear. In cell culture, taxane resistance usually occurs by upregulation of drug efflux pumps, but the relevance of this mechanism in patients is unclear, and P-glycoprotein inhibitors were not successful in the clinic [64,65]. An important finding, in both mice and patients, is that the response of drug-naive tumours to paclitaxel *did not correlate* with the extent of mitotic arrest shortly after drug administration [66,67]. This lack of correlation between a direct, cell-autonomous PD biomarker and tumour regression suggests that response variation is often due to variation in pathways that act downstream of mitotic perturbation, such as inflammatory signalling, more than to cell-autonomous factors such as tubulin isotype or P-glycoprotein expression. Recent work showed that cGAS and STING are required for senescence of pre-cancerous cells exposed to DNA damage or activated oncogenes, and proposed that progression to cancer involves downregulation or mutation of this pathway [52–54]. Selection for loss of cGAS/STING signalling during cancer progression might help explain patient-to-patient variation, and suggests biomarkers for predicting high responders. Finally, we propose that pharmacological amplification of residual cGAS/STING signalling will promote taxane responses, and combat acquired resistance. One possible target for such a drug is ENPP1, the only enzyme known to break down the cGAS product 2′3′cGAMP [68], but additional negative inputs could likely be identified and targeted.

## Data accessibility

This article has no additional data.
